# Cryptocurrency technology revolution: are Bitcoin prices and terrorist attacks related?

**DOI:** 10.1186/s40854-022-00445-3

**Published:** 2023-01-21

**Authors:** Yu Song, Bo Chen, Xin-Yi Wang

**Affiliations:** Institute of Defense Economics and Management, Central University of Finance and Economics, 39 Xueyuan South Road, Beijing, China

**Keywords:** Terrorist incident, Terrorist brutality, Bitcoin price, Time-varying, C32, G12, H56

## Abstract

As a financial innovation of the information age, cryptocurrency is a complex concept with clear advantages and disadvantages and is worthy of discussion. Exploring from a terrorism perspective, this study uses the time-varying parameter/stochastic volatility vector autoregression model to explore the risk hedging and terrorist financing capabilities of Bitcoin. Empirical results show that both terrorist incidents and brutality may explain Bitcoin price, but their effects are slightly different. Compared to terrorist brutality, terrorist incidents have a weaker impact on Bitcoin price, showing that Bitcoin investors are more concerned about the number of deaths than the frequency of terrorist attacks. In turn, the impact of Bitcoin price on terrorist attacks is negligible. Bitcoin is a potential means of financing terrorism, but it does not currently play an important role. Our research findings can help investors analyze and predict Bitcoin prices and help improve the theoretical system of anti-terrorist financing, helping to maintain world peace and security.

## Introduction

Bitcoin is a type of cryptocurrency with a decentralized transmission mode based on cryptography and blockchain technology (Majumder et al. 2019). Currently, Bitcoin has the highest market capitalization among cryptocurrencies, which has aroused the interest of both investors and policymakers (Grant and Hogan 2015; Li et al. 2021). Recently, the price of digital currency represented by Bitcoin has reached new highs. Owing to decentralization and anonymous trading, Bitcoin has shown potential as a hedge against the financial market and geopolitical risks (Bouoiyour et al. 2019; Aharon et al. 2021). Bitcoin price is not affected by market supply and demand but mainly fluctuates according to short-term geopolitical factors (Al-Mamun et al. 2020). As the global economy develops and global political changes, terrorism has become a serious problem, with terrorist attacks becoming more frequent. In some cases, the damage caused by terrorist activities has exceeded that of local wars and has become the main source of geopolitical risks (Orbaneja et al. 2018). Terrorist attacks seriously threaten people’s property and lives and create violent financial market fluctuations. Although the impacts of terrorist attacks on the stock market have been studied extensively (El Ouadghiri and Peillex 2018; Aksoy and Demiralay 2019; Markoulis and Katsikides 2020), the interaction between terrorist attacks and Bitcoin price cannot be eliminated.

Bitcoin may be used to finance terrorism. Owing to the decentralization and anonymity features of cryptocurrencies, illegal transactions cannot be regulated. Bitcoin is often associated with criminal activities, including money laundering and the financing of terrorism (Carmona 2015; Majumder et al. 2019). The Federal Reserve and European Central Bank have stated that Bitcoin is a highly speculative asset used in illegal financing activities (e.g., money laundering). Hence, a global regulatory consensus on Bitcoin is required. However, improving regulatory capacity usually lags behind financial technology innovation, providing new opportunities to finance terrorism. With the continuous development of internet technology, virtual currency, online payment, and dark web transactions have attracted great attention from terrorist organizations. International terrorist organizations, including the “Islamic State” (IS), use emerging technologies (e.g., the blockchain), to raise and transfer illegal funds (Weimann 2016; Majumder et al. 2019). In October 2015, the Financial Action Task Force on Money Laundering released a report entitled “Emerging Terrorism Financing Risks,” which specifically analyzed the terrorist financing risks of virtual currency. This report highlighted that virtual currencies, represented by Bitcoin, have increasingly become “accomplices” to various illegal and criminal activities, facilitating money laundering and terrorist financing. Terrorist financing based on emerging technologies can allow terrorist organizations to instantly transfer funds worldwide (Grant and Hogan 2015). However, it also increases the difficulty of counterterrorist financing in relevant countries (Irwin and Milad 2016; Patel and Pereira 2021). In the cryptocurrency technological revolution and spread of terrorism, studying the hedging properties and terrorist financing capabilities of Bitcoin is necessary. This study mainly addresses the following problems: Are terrorist attacks important drivers or factors of Bitcoin price fluctuations or price formation? To what extent can Bitcoin develop and morph into a currency utilized to finance terrorism? Does the interaction mechanism between Bitcoin price and terrorist attacks change over time?

This study makes the following contributions. First, it enriches the literature on the interaction between terrorist attacks and Bitcoin prices. In measuring the terrorist attack variable, we consider three measurement methods: terrorist incidents, terrorist brutality, and terrorist attack index. Moreover, we examined the time-varying effects of severe political and economic uncertainty event shocks on the relationship between terrorist attacks and Bitcoin price. Second, Bitcoin price is affected by terrorist attacks worldwide. The accuracy of the results would be greatly reduced if only one region or country was considered. Hence, our research explores the interaction between Bitcoin price and terrorist attacks globally. Additionally, we use monthly rather than daily data. Although terrorist incidents only last for limited periods, the panic, uncertainty, and insecurity these events generate can last for a long time (Kollias et al. 2013). As terror attacks cascade through news and social discourses, monthly data allow control for longer lags (Patel and Pereira 2021). Third, accurately identifying the impact mechanism between terrorist attacks and Bitcoin price at distinct points in time during periods of heightened terrorist attacks consists of an effective defence. We use the time-varying parameter/stochastic volatility vector autoregression (TVP-SV-VAR) model, which allows for structural changes, to analyze the interaction between terrorist attacks and Bitcoin price from a time-varying perspective. Compared with the constant-coefficient approach, the estimated parameters and variance of the disturbance term under the TVP-SV-VAR model changed over time, allowing us to capture the dynamic characteristics of the parameters in this study. Finally, from a risk management perspective, our research provides useful and relevant information to market participants—traders, investors, and risk managers—to help ensure better asset allocation during unforeseen shocks and rising terrorism risks. From an anti-terrorism financing perspective, this study analyzes the international anti-terrorism financing mechanism from an empirical level and improves the theoretical system and research results of terrorism financing. Hence, our results will be highly significant in combating terrorism and safeguarding world security and countries’ developmental interests.

The remainder of this paper is organized as follows. “Literature review” section comprises a literature review. “Theoretical mechanism and research hypothesis” section outlines the interactive mechanism between terrorist attacks and Bitcoin price. “Data” section describes the data. “Empirical results and discussion” section provides the empirical results and discussion. “Conclusions” section presents the main conclusions of this study.

## Literature review

Since its creation in 2008, Bitcoin and blockchain have been researched topics of constant interest to academia and international investors (Zhu et al. 2017; Kumar and Ajaz 2019; Shahzad et al. 2021; Zhao 2021; Zhang et al. 2021; García-Corral et al. 2022; Lorenzo and Arroyo 2022). Xu et al. (2019) review the current academic research on blockchain, especially in business and economics. Sebastião and Godinho (2021) examine the profitability of cryptocurrency trading strategies devised on machine learning techniques (e.g., linear models, random forests, and support vector machines). By covering 146 research papers on various cryptocurrency trading aspects, Fang et al. (2022) provide a comprehensive survey of cryptocurrency trading research. Bitcoin price is highly volatile, with frequent and extreme rises or falls. Much of the literature explores the properties of Bitcoin and the uncertainty factors that determine its price (Nasir et al. 2019; Jalali and Heidari 2020; Mensi et al. 2021; Neslihanoglu 2021; Almaqableh et al. 2022b). The relevant literature can be grouped into four themes as follows.

First, Bitcoin has a hedge and safe-haven properties. Fang et al. (2019) reveal that the uncertainty of global policy negatively and significantly impacts Bitcoin bond correlation and positively impacts correlations between Bitcoin equities and Bitcoin commodities. Hence, the possibility that Bitcoin acts as a hedge under specific uncertainty conditions. Aysan et al. (2019) examine the ability of geopolitical risks to predict the returns and volatility of Bitcoin. Hence, as global geopolitical risks continue to increase, investors should also use Bitcoin to hedge against uncertain risks. Plakandaras et al. (2021) examine whether information on the US–China trade war could be used to forecast Bitcoin returns. Their results suggest that future Bitcoin returns are unaffected by trade-related uncertainties, and investors can use Bitcoin as a safe haven in this context. Selmi et al. (2022) find that when risk is high, Bitcoin responds positively to the composite geopolitical risk indicator. Thus, Bitcoin can act as a safe haven for assets whose valuations plummet during violent geopolitical conflicts. However, some studies suggest that Bitcoin does not have risk-hedging properties. Bouri et al. (2017) indicate that Bitcoin cannot hedge risks but can be used in asset diversification. Bouoiyour and Selmi (2019) also indicate that Bitcoin may face many obstacles in becoming a safe-haven asset owing to the lack of regulation and excessive speculation. Generally, there is no consensus on whether Bitcoin can hedge market risk.

Second, Bitcoin can be a diversification option for investment portfolios. Investors are always examining alternative investment instruments as part of diversified investment portfolios. In recent years, the number of financial institutions including cryptocurrencies in their portfolios has accelerated (Fang et al. 2022). Bitcoin may offer diversification benefits for investors owing to its high average return and low correlation with financial assets (Guesmi et al. 2019). Eisl et al. (2015) highlight the impact of Bitcoin investment on an already well-diversified investment portfolio and indicate that Bitcoin should be included in optimal portfolios. Briere et al. (2015) confirm that Bitcoin investment offers significant diversification benefits. They show that including even a small proportion of Bitcoin may dramatically improve the risk-return trade-off of well-diversified portfolios. Carpenter (2016) demonstrate that Bitcoin can be a viable diversification tool, but its investment appeal may be skewed by return activity that occurred during a speculative bubble in 2013. Bouri et al. (2020a) suggest that Bitcoin can become a viable alternative to the conventional stock market considering the heightened trade policy-related uncertainties and provide diversification benefits for investors. Akhtaruzzaman et al. (2020) analyze portfolio diversification by adding Bitcoin to global industry portfolios. Results demonstrate that investment in Bitcoin provides an efficient diversification option for many industrial sectors and bonds. Qarni and Gulzar (2021) demonstrate that Bitcoin can provide significant portfolio diversification benefits for alternative currency foreign exchange portfolios.

Third, terrorism or geopolitical risk has an impact on Bitcoin price. Owing to the intensification of global geopolitical risks and frequent terrorist attacks, many scholars explain asset pricing from terrorism or geopolitical uncertainty perspectives (Salisu et al. 2022). Al-Mamun et al. (2020) show that geopolitical risk associated with acts of terrorism is the most consistent and significant positive determinant of Bitcoin volatility and risk premia. Bouri et al. (2020b) show that the price behavior of all cryptocurrencies is jumpy but only jumps in Bitcoin price are dependent on jumps in the geopolitical risk index. Patel and Pereira (2021) show that terrorist attacks lead to a downward trend in the macroeconomy, and pessimistic expectations of the future investment environment reduce Bitcoin returns. Almaqableh et al. (2022a) indicate that while terrorist attacks positively contribute to cryptocurrency returns, they also result in short-term risk shifting behavior for different cryptocurrencies.

Fourth, economic events impact Bitcoin price. Kyriazis (2020) concludes that the panic of market investment sentiment and various political and economic uncertainty events explain abnormal Bitcoin price fluctuations. Mokni et al. (2020) showed that an increase in economic policy uncertainty level is associated with an increase in the optimal weight of Bitcoin in a portfolio before the Bitcoin crash. However, economic policy uncertainty exerts a negative (positive) impact on the hedging ratio during low (high) uncertainty levels after a Bitcoin crash. Entrop et al. (2020) find that higher news—based Bitcoin sentiment affects the Bitcoin price of the futures market; conversely, attention and macroeconomic news have no impact on Bitcoin price discovery. Mokni (2021) investigated the predictive power of economic policy uncertainty on Bitcoin price volatility and emphasize that economic policy uncertainty can predict volatility only when the Bitcoin market is bullish. Wüstenfeld and Geldner (2021) analyze the dynamics between Bitcoin trading, price activities, and economic surprise shocks. Their results show that local and global shocks affect local Bitcoin activities and trading volatilities, confirming that economic events affect Bitcoin markets. Finally, Wu et al. (2022) show that global economic policy uncertainty positively influences Bitcoin returns but negatively impacts Bitcoin long-term volatility.

Besides studying the impact of terrorist attacks on Bitcoin price, studies have explored whether Bitcoin funds terrorist attacks from the terrorist financing perspective. As governments focus on taxation and their ability to control currencies, Bitcoin has become an important way to fund criminal acts and terrorism (Carmona 2015). The Federal Bureau of Investigation investigated the dangers of Bitcoin in terrorism financing. Their conclusions indicate that Iran uses Bitcoin to fund Hamas. The US Department of Justice confiscated $2 million worth of cryptocurrency from terrorist organizations in the Middle East, which al-Qaeda could have used to purchase weapons and train potential soldiers (U.S. Department of Justice 2020). Carmona (2015) showed that the anonymity and high efficiency of Bitcoin make the fund flow of terrorist organizations fast and opaque, posing a serious challenge to international counterterrorist financing. Weimann (2016) finds that terrorist organizations mainly use cryptocurrency to raise funds to receive Bitcoin donations, which can be used to buy weapons on the black market. Irwin and Milad (2016) demonstrate that terrorist organizations attempt to understand new and evolving digital and cryptocurrency technologies that may be used to move funds to areas where they are needed in a relatively anonymous and safe manner. Majumder et al. (2019) observe that this feature of anonymity, confidentiality, and lack of central surveillance made cryptocurrency a potential source for terrorism financing. Shah (2020) finds that Bitcoin price fluctuations are directly linked to terrorist attacks in countries with ISIS presence. Lee and Choi (2021) also find that terrorist attacks change with Bitcoin price. Simultaneously, the popularity of extortion software that uses Bitcoin as a ransom also affects the occurrence of terrorist activities. In summary, cryptocurrencies posing a potential money laundering and terrorism financing threat have found a clear consensus.

## Theoretical mechanism and research hypothesis

### Mechanism of the impact of terrorist attacks on Bitcoin price

Uncertainty about terrorist attacks may be the key element that significantly impacts the Bitcoin investment climate (Patel and Pereira 2021). When terrorist attacks trigger serious market panic, greater fluctuations in geopolitics, macroeconomics, and financial markets occur. Owing to the decentralization, security, and anonymity associated with the rise of Bitcoin, an increasing number of investors and institutions have become interested in digital currency (Yousaf and Ali 2020). In the early days of Bitcoin, its performance was better than that of gold, and its price remained stable for a long time. More investors are admitting that Bitcoin can evolve into a major safe-haven asset (Qarni and Gulzar 2021). Hence, the Bitcoin market may potentially attract an inflow of safe-haven funds, showing a more significant growth rate than the gold market (Al-Mamun et al. 2020). Some investors call it “digital gold” (Huynh et al. 2020).

While the early consensus on Bitcoin is clear, it is far from being “digital gold.” Particularly, in recent years, Bitcoin price has fluctuated greatly, and it has neither shown an inverse relationship with the global stock market nor followed the price trend of gold and bonds (Mokni et al. 2020). Conversely, terrorist attacks have increasingly shown a negative correlation with Bitcoin price, as Bitcoin has sometimes failed to demonstrate its safe-haven asset characteristics. The main transmission mechanisms of this effect may be as follows. On the one hand, terrorist attacks cause market turmoil and increased uncertainty. In a crisis, the concept of “cash is king” prompts Bitcoin investors to sell in large quantities, causing further panic selling (Baele et al. 2020). Conversely, terrorist attacks lower Bitcoin prices through a decline in the short-term macroeconomic cycle. When a pessimistic investment environment spreads, investors focus on the safety and liquidity of holding physical assets and are more inclined to store assets with less volatility to reduce future losses (Patel and Pereira 2021). In summary, the frequent and substantial fluctuations of Bitcoin in recent years have reduced investor confidence, and people are no longer inclined to regard it as a safe-haven asset. Thus, we propose the following hypothesis:

#### Hypothesis I

Terrorist attacks are critical factors leading to Bitcoin price fluctuations. However, in the early and later stages of Bitcoin, its ability to avoid terrorism risks are opposed.

### Mechanism of Bitcoin price impacts on terrorist attacks

Most of the world’s well-known terrorist organizations derive their income mainly from oil trafficking, smuggling, and so on (Lee 2018; Ghatak and Karakaya 2021). However, as anti-terrorism efforts strengthen in various countries, these traditional projects are becoming increasingly difficult to implement. Simultaneously, once these terrorist organizations realized that money deposited in the bank and their withdrawals could easily be traced, they explored new ways to raise funds, such as through Bitcoin (Weimann 2016; Kfir 2020).

Terrorist organizations favor Bitcoin owing to the following three main reasons. First, cryptocurrency is generally characterized by the anonymity of transactions and the free cross-border flow of funds. Hence, terrorists can easily conceal the source and location history of their funds. Moreover, terrorists will find circumventing foreign exchange quotas and regulations on foreign exchange remittances abroad easy. This brings convenience to money laundering, terrorist financing, and capital control evasion (Carmona 2015; Patel and Pereira 2021). Second, as the identity of the Bitcoin owner is encrypted in the Bitcoin network, people can only identify the source, flow direction, and circulation mode of Bitcoin. However, the identity of the Bitcoin owner cannot be known. Moreover, the government cannot regulate Bitcoin transactions owing to this characteristic (Irwin and Milad 2016). Third, the “convertibility” of Bitcoin allows terrorists to turn cryptocurrency into real currency. Simultaneously, the decentralized nature of Bitcoin also provides an opportunity for terrorists to escape bank and government supervision, enabling them to quickly transfer funds without formal financial institutions knowing (Irwin and Turner 2018; Lee and Choi 2021).

Owing to these advantages, Bitcoin can support terrorist network financial support systems, rendering the traditional and most effective counterterrorism policies completely outdated (Carmona 2015). Worldwide, terrorists are gradually using Bitcoin as a payment carrier for terrorist activities, online money laundering, blackmail, and other illegal and criminal activities (Pflaum and Hateley 2013). As terrorist organizations begin using Bitcoin to raise funds, changes in Bitcoin price will affect the ability of terrorist organizations to raise funds, affecting their ability to launch terrorist attacks. Specifically, on the one hand, the rising Bitcoin price will make the Bitcoin held by terrorist organizations more valuable, providing them with sufficient funds to launch more serious terrorist attacks. On the other hand, rising Bitcoin prices will encourage terrorist organizations to bypass the surveillance mechanism of the banking system of the respective countries to anonymously finance terrorist attacks (Majumder et al. 2019). This effort is usually worthwhile as a high Bitcoin price is more beneficial and efficient for terrorist financing. Therefore, in the Granger sense, Bitcoin price changes may occur before terrorist attacks.

Despite the growing connection between Bitcoin and terrorist attacks, some studies show no large-scale use of cryptocurrency by terrorist organizations to raise or transfer funds (Irwin and Milad 2016; US Department of the Treasury 2018). Most terrorist organizations operate in remote mountainous or rural areas with poor infrastructure and technology levels. Terrorists cannot use advanced computer technology to mine or trade Bitcoin. Moreover, terrorists may not be able to convert or trade Bitcoin for real currency (Irwin and Milad 2016). While this terrorist financing method has not been applied globally, it is the “new favorite” of terrorist organizations. Bitcoin’s status in terrorist activities has gradually improved. For example, existing studies have shown that many terrorist organizations have mined Bitcoin by outsourcing, compensating for their technical disadvantages. Bitcoin represents an important financial option for terrorist networks as it can almost seamlessly integrate into existing funding frameworks (Carmona 2015). Thus, we propose the following hypothesis:

#### Hypothesis II

Bitcoin is a potential means of terrorist financing but cannot play an important role.

### Dynamic interaction between Bitcoin prices and terrorist attacks

Although terrorism risk will affect Bitcoin prices and cryptocurrencies pose significant money laundering and terrorism financing threats, whether this interaction is static or dynamic must be determined before an accurate picture of the risks associated with this emerging and potentially revolutionary technology can be obtained. Interaction between Bitcoin prices and terrorist attacks is evidently heterogeneous over time for the following reasons.

First, terrorism risk changes over time, depending on where terrorist attacks occur, why they occur, and the areas they affect. This phenomenon leads to different impact directions, intensities, and duration of terrorist attacks on the Bitcoin market reflected in the different price fluctuations of Bitcoin. Second, Bitcoin price is characterized by strong volatility and a large fluctuation range. The performance of the Bitcoin market is also significantly different under different terrorist attacks. These characteristics make relationships between terrorist attacks and Bitcoin prices complex and dynamic. Third, as terrorist attacks become the norm, their impact on Bitcoin prices can weaken and change with the extension of time. Hence, the relationship between the two will not be as closely linked as in the past, and the influence of terrorist attacks may become mostly nonlinear, with greater uncertainty and time delays.

Additionally, when analyzing the relationship between terrorist attacks and Bitcoin price, the existing literature analyzes terrorism purely as an event. However, the interaction between Bitcoin prices and terrorist attacks is quite complex and also determined by other various complicated factors (e.g., severe political and economic uncertainty events). Different types of uncertainty events can have a knock-on effect on the interaction between terrorist attacks and Bitcoin prices. Hence, exploring the effects of contingencies in the context of political and economic uncertainty risks is necessary. Therefore, we propose the following hypothesis:

#### Hypothesis III

The interaction between Bitcoin prices and terrorist attacks changes over time and is subject to economic or political uncertainty.

## Data

We selected the sample period of October 2013 to December 2020 to assess the interaction between terrorist attacks and Bitcoin price, with start and end dates purely contingent on data availability. The CoinDesk Bitcoin Price Index (XBP) provides the most accurate Bitcoin price. It represents the average Bitcoin price across all major international Bitcoin exchanges that meet the criteria specified by the XBP and compares it to the price on another selected exchange (Al-Yahyaee et al. 2019). We obtained data on terrorist attacks from the Global Terrorism Database at the University of Maryland (Miao et al. 2017; Huang et al. 2017; Lee and Choi 2021). This variable is defined as the number of terrorist attacks experienced by countries worldwide in a given month (Lee 2018; Patel and Pereira 2021). Additionally, terrorist attacks have a destructive influence on the Bitcoin price depending on the brutality of the attack. Hence, we also use the number of fatalities as a proxy for the brutality of a terrorist attack (Frey et al. 2007; Orbaneja et al. 2018). To distinguish between these two aspects of terrorist attacks, we measure terrorist incidents (number of terrorist attacks per month) and terrorist brutality (number of fatalities per attack).

Besides considering the impact of terrorist attacks on Bitcoin price, we also examine the role of the US dollar index (USDX). In the current and future settings, any geopolitical events, especially terrorist attacks, can affect dollar value (Su et al. 2020a). As terrorist attacks may affect the development of the world economy, any interest rate increase or reduction policy adopted by the Federal Reserve relative to changes in the economic situation will directly affect the USDX. Hence, any fluctuations in Bitcoin price may be influenced by the US dollar as the US dollar is considered the denomination currency of Bitcoin. Hence, if the value of the US dollar declines, it will lead to a rise in the Bitcoin price. Moreover, if the US dollar appreciates, it will cause a decline in the Bitcoin price. Therefore, the USDX may affect the mutual influence between terrorist attacks and Bitcoin price. USDX is a comprehensive index representing the value of the US dollar against a basket of foreign currencies (Dey et al. 2020).

**Fig. 1:**
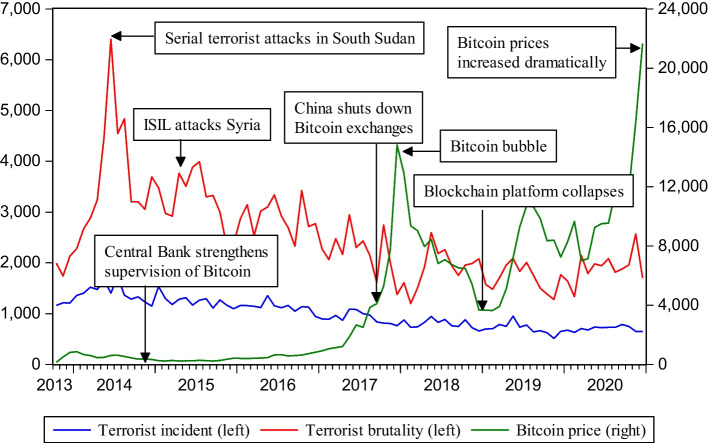
The trends of terrorist incidents, terrorist brutality, and Bitcoin price

Figure 1 shows that Bitcoin has been extremely volatile, especially during the extreme bull market and abrupt bear market in 2017 and 2018, respectively. In 2013, Germany recognized Bitcoin as a private currency. Meanwhile, China’s interest in Bitcoin has continued rising, with many investors pouring into the Bitcoin market, and Bitcoin price exceeded 100 dollars. From 2014 to 2015, the Bitcoin price began falling again, caused by China’s policy of prohibiting banks and payment institutions from trading Bitcoin. In 2016, owing to the halving of Bitcoin’s annual output and Brexit, investors are increasingly entering the Bitcoin market, some investment institutions also invested in Bitcoin, and more merchants and companies accepted Bitcoin as payment. In December 2016, the unit price of Bitcoin exceeded US$1,000. In 2017, Bitcoin price continued rising by as much as 1,700% throughout the year, and Bitcoin reached its highest price. Many speculators and investment institutions flowed into the Bitcoin market, and Bitcoin entered a crazy bull market. However, in 2018, as China banned Bitcoin trading completely and global regulation tightened, Bitcoin prices entered a downward trend. In 2019, a new rebound trend occurred for chasing risks in the global market, and huge amounts of venture capital flowed into the Bitcoin market. The rapid development of blockchain technology and policy support has also provided a favorable environment for the Bitcoin market. In 2020, the COVID-19 outbreak led to a global economic downturn, and investors’ risk aversion caused Bitcoin prices to soar. Generally, since Bitcoin was created, the price trend has both skyrocketed and plummeted. Arguably, Bitcoin price is more volatile than terrorist attacks. Regardless, terrorist attacks and Bitcoin prices do not always have a consistent relationship throughout the sample period and show complex interactions in different periods.

Table 1 shows the descriptive statistics for terrorist incidents, terrorist brutality, Bitcoin price, and the USDX, and averages for these variables were 995.644, 2492.011, 4321.573, and 102.725, respectively. The standard deviation of terrorist brutality is greater than that of terrorist incidents, indicating greater volatility. The skewness of USDX is less than 0, meaning that its data distribution is a “negative skew distribution” compared with the normal distribution. The skewness of the other three variables is greater than 0, indicating that they are “positive skew distributions.” Kurtosis of terrorist incidents and the USDX is below 3.0, indicating that these variables have platykurtic or “thin-tailed” distributions. In contrast, kurtosis for terrorist brutality and Bitcoin price reveals a leptokurtic or “fat-tailed” distribution. The Jarque–Bera test rejects the null hypothesis that variables are normally distributed at the 1% significance level. This indicates that terrorist brutality, Bitcoin price, and the USDX variables have non-normal distributions. All variables are transformed into logarithmic values to eliminate heteroscedasticity in time series. Further, to obtain a stationary time series, we perform a first-order difference on the data.

**Table 1:** Descriptive statistics for terrorist incidents, terrorist brutality, Bitcoin price, and the USDX

	Terrorist incidents	Terrorist brutality	Bitcoin price	USDX
Mean	995.644	2492.011	4321.573	102.725
Median	911.000	2275.000	1870.707	104.682
Maximum	1726.000	6405.000	21,657.070	113.425
Minimum	512.000	1199.000	149.456	88.269
Standard deviation	277.853	892.650	4655.892	6.467
Skewness	0.433	1.423	1.101	− 0.980
Kurtosis	2.296	6.232	3.920	2.983
Jarque–Bera	4.509	67.218***	20.648***	13.936***

The unit of the Bitcoin price is in dollars

***Denotes significance at the 1% level

## Empirical results and discussion

For comparative purposes, we employed several unit root tests, namely, the Augmented Dickey-Fuller (ADF), Phillips and Perron (PP), and Kwiatkowski-Phillips-Schmidt-Shin (KPSS) tests to test data stability. Table 2 results demonstrate that the ADF and PP tests reject the null hypothesis of non-stationarity for the underlying series. The KPSS test accepts the null hypothesis of stationarity for the underlying series. Accordingly, results derived from the unit root tests establish that all the series are stationary. The optimal lag length selected based on the Schwarz Information Criteria was 1.

**Table 2:** Result of the unit root test

	Unit root test		
Variables	ADF	PP	KPSS
Terrorist incidents	− 9.079***	− 14.537***	0.026
Terrorist brutality	− 11.937***	− 12.539***	0.113
Bitcoin price	− 7.864***	− 7.867***	0.068
USDX	− 5.416***	− 5.013***	0.052

***Denotes significance at 1% levels

Table 3 shows that the mean of the estimation results of the parameters is within the 95% confidence interval. Geweke (1992) refers to convergence diagnostics, which are less than the 5% critical value and indicates that the null hypothesis of convergence to the posterior distribution cannot be rejected. Moreover, the maximum value of the inefficiency factors is 74.83, enough to make the Markov chain converge. Thus, the estimation of the TVP-SV-VAR model is valid.

**Table 3:** Parameter estimation results of the model

Parameters	Mean	Standard deviation	95% confidence interval	Geweke’s Z-score	Inefficiency factors
$(\sum_{\beta } )_{1}$	0.023	0.003	(0.018, 0.029)	0.173	7.14
$(\sum_{\beta } )_{2}$	0.023	0.003	(0.018, 0.029)	0.908	3.18
$(\sum_{a} )_{1}$	0.084	0.036	(0.041, 0.176)	0.248	55.02
$(\sum_{a} )_{2}$	0.094	0.039	(0.044, 0.190)	0.780	39.18
$(\sum_{h} )_{1}$	0.127	0.060	(0.054, 0.285)	0.252	47.46
$(\sum_{h} )_{2}$	0.144	0.066	(0.059, 0.315)	0.025	74.83

The parameters are the posterior estimation of the first two diagonal elements of $\sum_{\beta }$, $\sum_{a}$, and $\sum_{h}$, and the results of the remaining elements also fulfill the statistical requirements

The 5% critical value of Geweke is 1.96

**Fig. 2:**
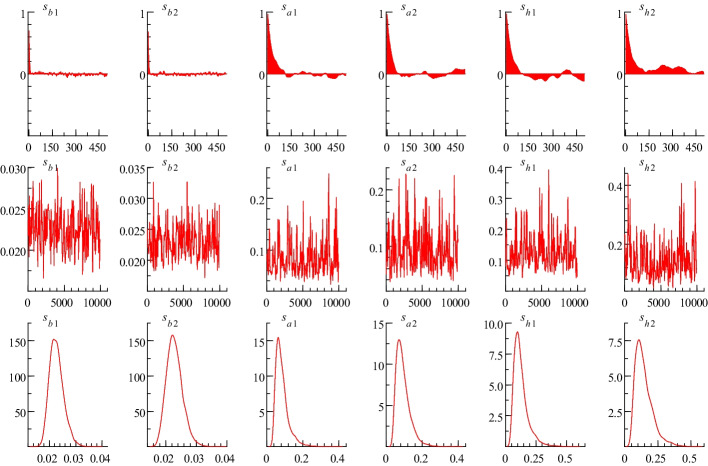
Sample autocorrelations, paths, and posterior densities of Markov Chain Monte Carlo estimation

Figure 2 presents the sample autocorrelations, paths, and posterior densities of the Markov chain Monte Carlo estimation results. Coefficients decrease to zero (in the first row), which means that no obvious autocorrelation in the samples can be found. The trajectory fluctuates around the mean (in the second row), indicating that no obvious trend can be found in the sample. Samples converge to the posterior distribution (in the third row). Thus, sampling results are robust, with no obvious divergence, and dynamic influences among these variables can be further explored by applying the TVP-SV-VAR model.

**Fig. 3:**
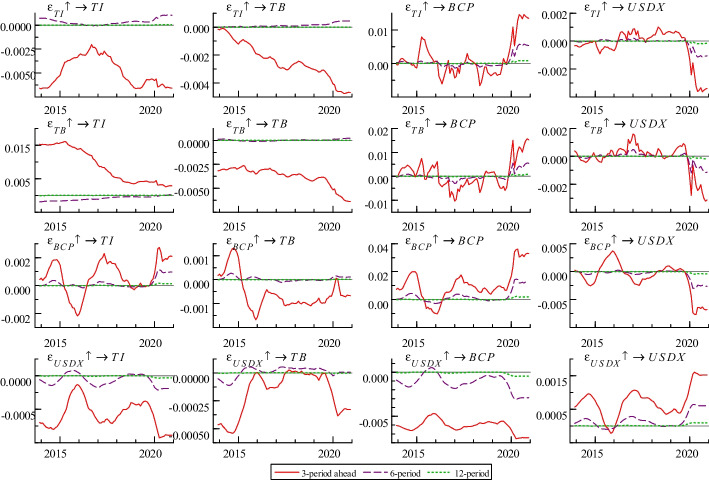
Impulse responses at different horizons

Figure 3 highlights impulse responses for different horizons (3, 6, and 12 months). As in the third column, the impacts of these variables on Bitcoin price are close to 0 at the 6- and 12-month horizons. However, the impacts are unstable at the 3-month horizon, and the influence direction and degree of each variable on Bitcoin price differ. Bitcoin price is affected by that of the previous period. Overall, the effect is mostly positive (except for a few months around 2016), which indicates that the current high Bitcoin price generates the expectation of future prices rising continuously, pushing up the future Bitcoin price.

Two measurement dimensions of terrorist attacks—terrorist incidents and terrorist brutality—have time-varying effects on Bitcoin price, mainly manifesting in positive (before 2016 and after 2020) and negative correlation (2016–2020).^1^ Hence, we should observe the interaction from a dynamic perspective. This time-varying phenomenon not only proves Hypothesis III but is also the same as many previous studies (e.g., Bouri et al. (2021)) which find that the return equicorrelation in the cryptocurrency market is very time-varying and unstable. Mokni et al. (2020) also show that uncertainty risk has a different impact on the Bitcoin price before and after the Bitcoin crash.

Before 2016, a similar trend occurred wherein terrorist attacks seemed to boost Bitcoin prices. The main reason for this is that Bitcoin price is stable, and volatility is quite low before 2016. Investors use Bitcoin to hedge against terrorism risk. Bitcoin’s investment attractiveness is reflected in its increasing acceptance and trust (Narayan et al. 2018). Moreover, the amount of Bitcoin is scarce compared with other currencies, and this scarcity may make Bitcoin a safe-haven asset in the future, increasing investor confidence in its security (Su et al. 2020b). Since the outbreak of COVID-19 in 2020, the Federal Reserve has injected a large amount of dollar liquidity into the market to solve the liquidity crisis and stimulate the economy, causing huge fluctuations in global financial markets, including digital currencies. Meanwhile, traditional safe-haven assets such as the US dollar and gold have underperformed during COVID-19. Moreover, people tend to use Bitcoin to hedge against terrorism risk. Hence, results before 2016 and after 2020 may indicate that Bitcoin is considered a safe-haven asset.

From 2016 to 2020, the negative correlation between terrorist attacks and Bitcoin price proves that the demand for risk hedging will not increase the demand and Bitcoin price when terrorist attacks spread panic. This is because, by holding Bitcoin, sharp fluctuation in Bitcoin price after 2016 may have made investors lose confidence and no longer want to deal with terrorism risk. The main transmission mechanisms of this effect may be as follows. An increase in terrorist attacks makes the geopolitical environment unstable, the panic mood of investors rises, and the demand for risk avoidance is relatively high. Moreover, they may be more focused on the safety of holding assets, causing speculators to transfer part of their funds from risky assets (e.g., Bitcoin) to traditional safe-haven assets (gold and US dollars). During this period, investors no longer held Bitcoin, several speculators and investment institutions left the Bitcoin market, and declining demand drove down Bitcoin prices down. Hence, the results of this period show that Bitcoin is a highly speculative asset, does not conform to the properties of the general currency, and should not be used in asset diversification or risk management (Ciaian et al. 2016; Bouoiyour and Selmi 2019). As Bundesbank stated, Bitcoin is more of a venture capital asset than a currency. Moreover, the above results can also partially support hypothesis I. However, in the early and later stages of Bitcoin, its ability to avoid terrorism risks are opposed.

By comparing terrorist incidents and brutality, we notice that terrorist incidents in Bitcoin price are weaker than that of terrorist brutality, especially after 2016. Hence, Bitcoin investors are more concerned about the number of deaths than the frequency of terrorist attacks. When a terrorist attack results in numerous deaths, panic spreads quickly to Bitcoin trading, thus affecting Bitcoin price. The impact of USDX on Bitcoin price is negative; however, compared with other factors, its impact is negligible (the coefficient is approximately − 0.005). This result remains consistent with Baur and Dimpfl (2021)’s study, showing that the dynamics of Bitcoin volatility are different from and unrelated to foreign exchange volatility. The reason a declining USDX makes Bitcoin price rise is that Bitcoin is priced in US dollars. Hence, the trend of the USDX directly affects Bitcoin’s price trend. To compensate for the real value of Bitcoin, Bitcoin price in US dollars should rise accordingly. Therefore, with the depreciation of the US dollar, international Bitcoin price generally shows an upward trend.

From the perspective of reverse causality (the third row of Fig. 3), Bitcoin price has a negligible impact on terrorist incidents and terrorist brutality, and the absolute value of the coefficient is less than 0.002 in most periods. The following three perspectives show that Bitcoin is not an effective means of financing terrorism. First, the conversion of Bitcoin into cash requires certain infrastructure and related technologies. Most areas where terrorists live do not have the infrastructure for managing cryptocurrency. Moreover, exchanging materials through Bitcoin (Irwin and Milad 2016) is almost impossible. Second, the lack of liquidity of a cryptocurrency is the biggest obstacle preventing them from being widely adopted. Governments heavily regulate many cryptocurrency exchanges (e.g., the global implementation of the “Know Your Customer” and “Anti-Money Laundering” regulatory provisions), which creates liquidity barriers. That is, terrorist organizations may find smoothly exchanging Bitcoin for cash difficult. Third, Bitcoin is not completely anonymous as anyone can use the Bitcoin public blockchain to track the flow of transactions. When terrorists intend to sell Bitcoin on a regulated exchange, government agencies can easily determine the identity of the Bitcoin user. Hence, terrorists cannot obtain Bitcoin without exposing the user’s identity. Finally, Bitcoin is highly volatile and may have bubbles. Terrorist organizations have to take speculative risks when using Bitcoin to raise funds (Carmona 2015). While Bitcoin is commonly used among cybercriminals, the main funds of terrorists still originate from banks and remittances (US Department of the Treasury 2018). Hence, the results in Fig. 3 show that although Bitcoin is a potential means of terrorist financing, it is currently insufficient for important roles. Therefore, hypothesis II is supported.

**Fig. 4:**
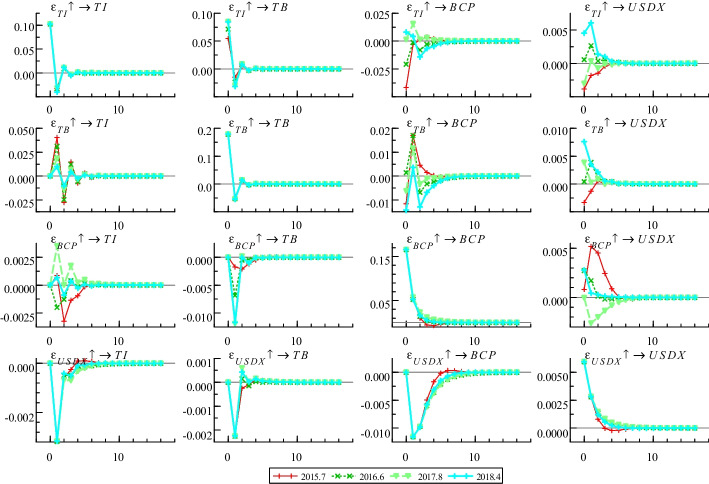
Impulse responses at different time points

Moreover, we also examined the effects of severe political and economic uncertainty event shocks on the relationship between terrorist attacks and Bitcoin price. We selected four representative economic and political uncertainty events. These emergencies not only have a serious impact Bitcoin market impact but also last longer, which can correspond to the monthly data frequency in this paper. In July 2015, Greece’s financial system collapsed and plunged into a debt crisis, affecting both the European and global economies. According to the statistics of the Bitcoin Trading Center, the trading volume of Bitcoin from Greece has increased significantly. In June 2016, Brexit considerably impacted many aspects of European integration and global geopolitics. Brexit brought a strong risk aversion to the global market, triggering a sharp increase in the demand for alternative assets, and Bitcoin price has risen by nearly 13%. In August 2017, US President Donald Trump signed an administrative memorandum, instructing the US trade representative Robert Lighthizer to launch an investigation into “China’s unfair trade practices” to ensure the protection of US intellectual property rights and technologies. Against increasing US economic policy uncertainty, Bitcoin was anticipated to become a “safe haven” before Trump’s trade war occurred. In April 2018, the US announced a precision strike against Syria, with Britain and France jointly participating in military operations against Syria. Syria was attacked by 110 Tomahawk missiles from Britain, France, and the US Bitcoin price, with its safe-haven properties and appreciation potential, has risen steadily as a result of the war in Syria. Figure 4 presents the result of impulse responses at different time points.

Figure 4 shows that serious economic or political uncertainty events interfere with the impact of terrorist attacks on the Bitcoin price, further supporting hypothesis III. Therefore, investors should not only examine the impact of terrorist attacks on the Bitcoin market but also the interference of various economic or political uncertainties on the financial market, and reasonably arrange any Bitcoin investment. Specifically, during the Greek debt crisis, terrorist attacks negatively affected Bitcoin price, but this rebounded in the subsequent several periods. Both terrorist incidents and terrorist brutality pulled the Bitcoin price down initially, with the role of terrorist brutality being stronger. During Brexit and the Sino-US trade war, terrorist incidents and terrorist brutality had opposite impacts on Bitcoin price. Terrorist incidents decreased Bitcoin price during Brexit, but they increased it during the Sino-US trade war. Terrorist brutality had the opposite effect, that is, the terrorist brutality increased Bitcoin price during Brexit, but they decreased it during the Sino-US trade war. This shows that investors were more inclined to use Bitcoin as a safe-haven asset to prevent the risk of terrorist incidents during the Sino-US trade war, and more inclined to use Bitcoin to guard against terrorist brutality during the Brexit period. During the Syrian war, except for the initial moments of terrorist incidents, terrorist incidents and terrorist brutality caused the Bitcoin price to fall not only because Bitcoin had no longer been considered a safe-haven asset in recent years but also as the market panic caused by the war was higher than for terrorist attacks. Therefore, the dual effects of war and terrorist attacks directly led to a decline in Bitcoin price.

**Fig. 5:**
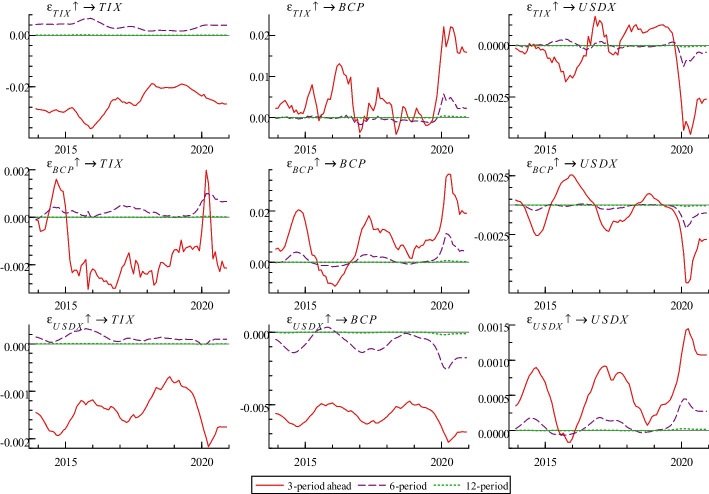
Robustness analyses (impulse responses at different horizons)

**Fig. 6:**
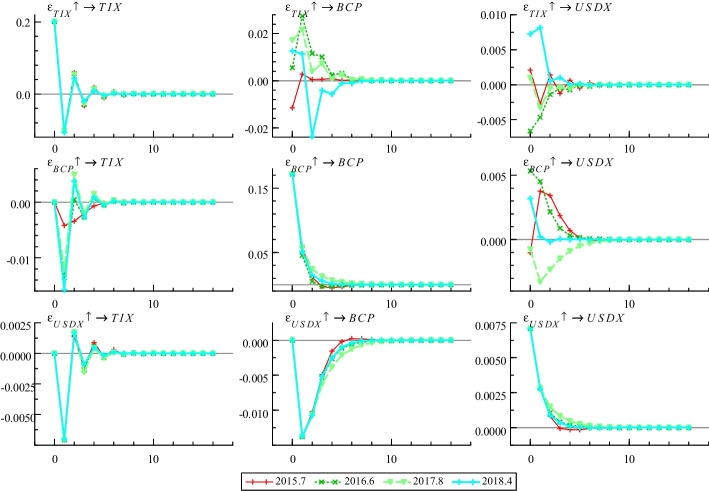
Robustness analyses (impulse responses at different time points)

To demonstrate the reliability of the empirical results, we created a terror index to replace terrorist incidents and terrorist brutality, perform the same analysis, and compare the results as a robustness check. Many previous studies have constructed terror indices by considering the number of victim fatalities, victim injuries, and terror events (Eckstein and Tsiddon (2004), Arin et al. (2008), Balcilar et al. (2017)). However, in addition to the above three indicators, the number of people taken hostage by terrorists should also be an important source for constructing the terror index. This is because when terrorists take hostages to demand ransoms, create panic, and blackmail the government, the volatility these events impose on the cryptocurrency market is similar to events wherein casualties are involved. Therefore, in this study, the terror index is defined as the natural logarithm of (*e* + number of people killed + number of people wounded + number of people taken as hostages + number of terrorist attacks) that occurred each month. Here *e* denotes the exponential function that occurred each month. This equation means that the index takes a value of 1 on months with no terror attacks. We follow the papers by Eckstein and Tsiddon (2004) in this regard. Figures 5 and 6 show the results of the robustness test. By observing Figs. 5 and 6, we can see that the relationship between the terror index and Bitcoin price is basically consistent with the previous research. Generally, the robustness test proves that the empirical results are reliable.

## Conclusions

As cryptocurrencies remain in the development phase, policymakers in developed countries and developing economies should be aware that terrorism risks can significantly affect Bitcoin price volatility. Simultaneously, interest in the association between terrorists using cryptocurrency to fund terrorist activities has gained traction in recent years. This study examined how terrorist attacks impact Bitcoin prices from October 2013 to December 2020. We aimed to reveal time-varying attributes at different horizons and time points by applying the TVP-SV-VAR model. Empirical results show that terrorist incidents and brutality are important factors affecting Bitcoin price, mainly manifesting in positive correlation (before 2016 and after 2020) and negative correlation (2016–2020). By comparing terrorist incidents and brutality, we notice that, unlike terrorist brutality, terrorist incidents have a weaker influence on Bitcoin price. Hence, Bitcoin investors are more concerned about the number of deaths than the frequency of terrorist attacks. We studied the reverse causal mechanism from Bitcoin price to terrorist attacks and found that Bitcoin price has negligible impact on the frequency or brutality of terrorist attacks. While Bitcoin is a potential means of financing terrorism, its role is not sufficient enough to be considered important. Moreover, we examined the effects of severe political and economic uncertainty event shocks on the relationship between terrorist attacks and Bitcoin price. We believe that investors should not only pay attention to the impact of terrorist attacks on the Bitcoin market but should also prioritize the interference of various economic or political uncertainties on the financial market, and reasonably arrange any Bitcoin investment.

The emergence of cryptocurrency results from market development driven by technological progress. The government should improve the regulatory mechanism while supporting Bitcoin development. Based on the above research, this study proposes the following policy recommendations. First, the impact of terrorist attacks on Bitcoin prices is time-varying and volatile. Hence, Bitcoin is a highly speculative asset and does not have risk-hedging properties. Investors should invest carefully and avoid related financial market risks and Bitcoin bubble bursts by diversifying their investment strategies. Second, the government and industries need to strengthen the security of network technologies and protocols to ensure the regular and orderly development of the cryptocurrency market. Relevant regulatory authorities should continue strictly supervising Bitcoin, investigating false news and malicious media speculation, and protecting investor interests. Third, Bitcoin regulation has not reached a consensus in the international community. This results in serious money laundering and speculation. While Bitcoin cannot adequately support terrorist activities, monitoring Bitcoin price and ransomware attacks are necessary. The government should issue laws and regulations related to taxation, anti-money laundering, payment, and so on. For example, anti-money laundering regulations should include a stipulation that traders are not allowed to conduct anonymous transactions in virtual currencies. Finally, countries should actively seek cooperation, share information and intelligence about global Bitcoin transactions, prohibit black market transactions, regulate global Bitcoin supervision, and create a unified regulatory framework.

Our research also has some limitations. Owing to data unavailability, we did not use the latest research data and only considered cryptocurrency as represented by Bitcoin. Future research should consider several major cryptocurrencies and, on this basis, explore the impact of geopolitical events or uncertainty risks other than terrorist attacks on the cryptocurrency portfolio.

## Data Availability

The datasets used during the current study are available from the corresponding author on reasonable request.

## Abbreviations

IS: Islamic State
TVP-SV-VAR: Time-varying parameter/stochastic volatility vector autoregression
U.S.: United States
XBP: CoinDesk Bitcoin Price Index
USDX: U.S. dollar index
COVID-19: Corona Virus Disease 2019
ADF: Augmented Dickey-Fuller
PP: Phillips and Perron
KPSS: Kwiatkowski-Phillips-Schmidt-Shin

## Footnotes

1 The differences in individual months are ignored, and we mainly consider the overall trend.
